# Supplementary material for: Impact of the COVID-19 Pandemic on the Incidence and Thickness of Cutaneous Melanoma in Belgium
Source: Biomedicines. 2023 Jun 6;11(6):1645. doi: 10.3390/biomedicines11061645 (PMC10295984; doi:10.3390/biomedicines11061645)
Supplement: Supplementary file 1 [file biomedicines-11-01645-s001.zip › biomedicines-2414601-supplementary.pdf]

Supplementary Data

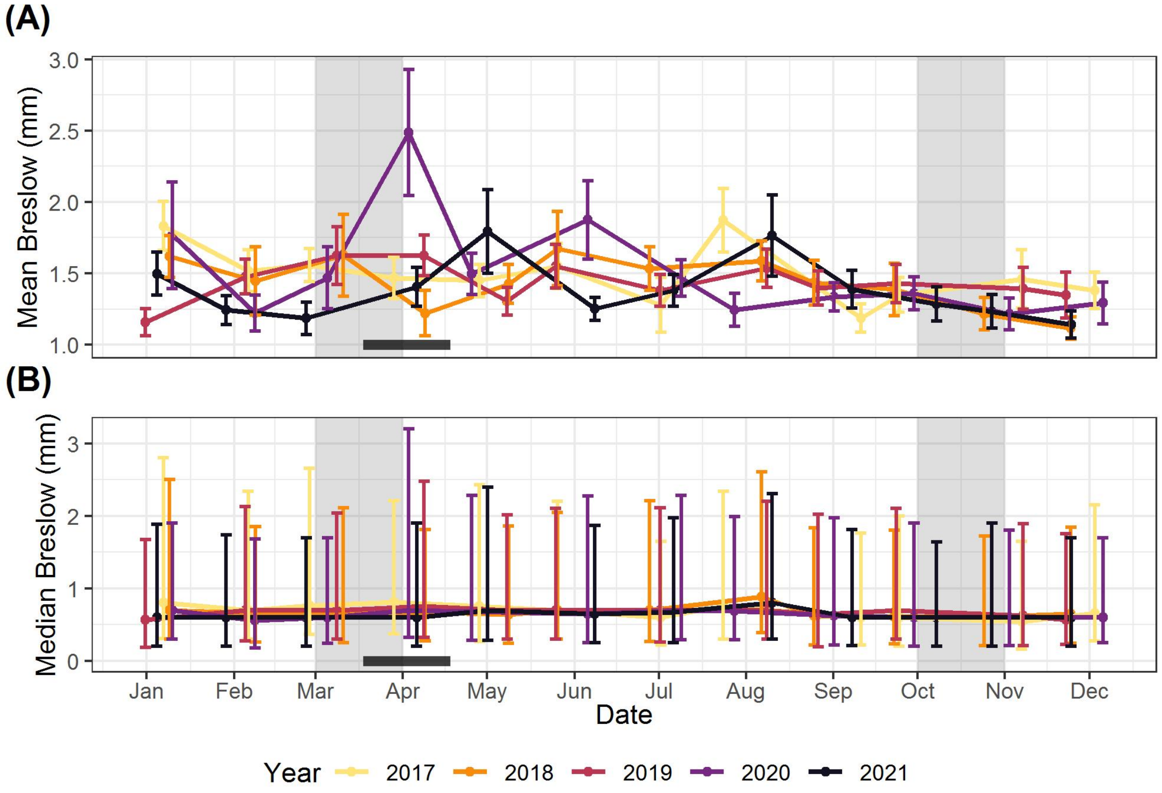

Figure S1. Evolution in monthly mean (a) and median (b) Breslow value for different registration years separately. 25th and 75th percentiles are shown with brackets. In (b) random jitter was added to x-axis values to improve clarity. The grey areas correspond to the periods in 2020 in which an exponential growth in the number of COVID-19 infections was observed; the bold black line corresponds to the period in 2020 in which the stringent lockdown took place.

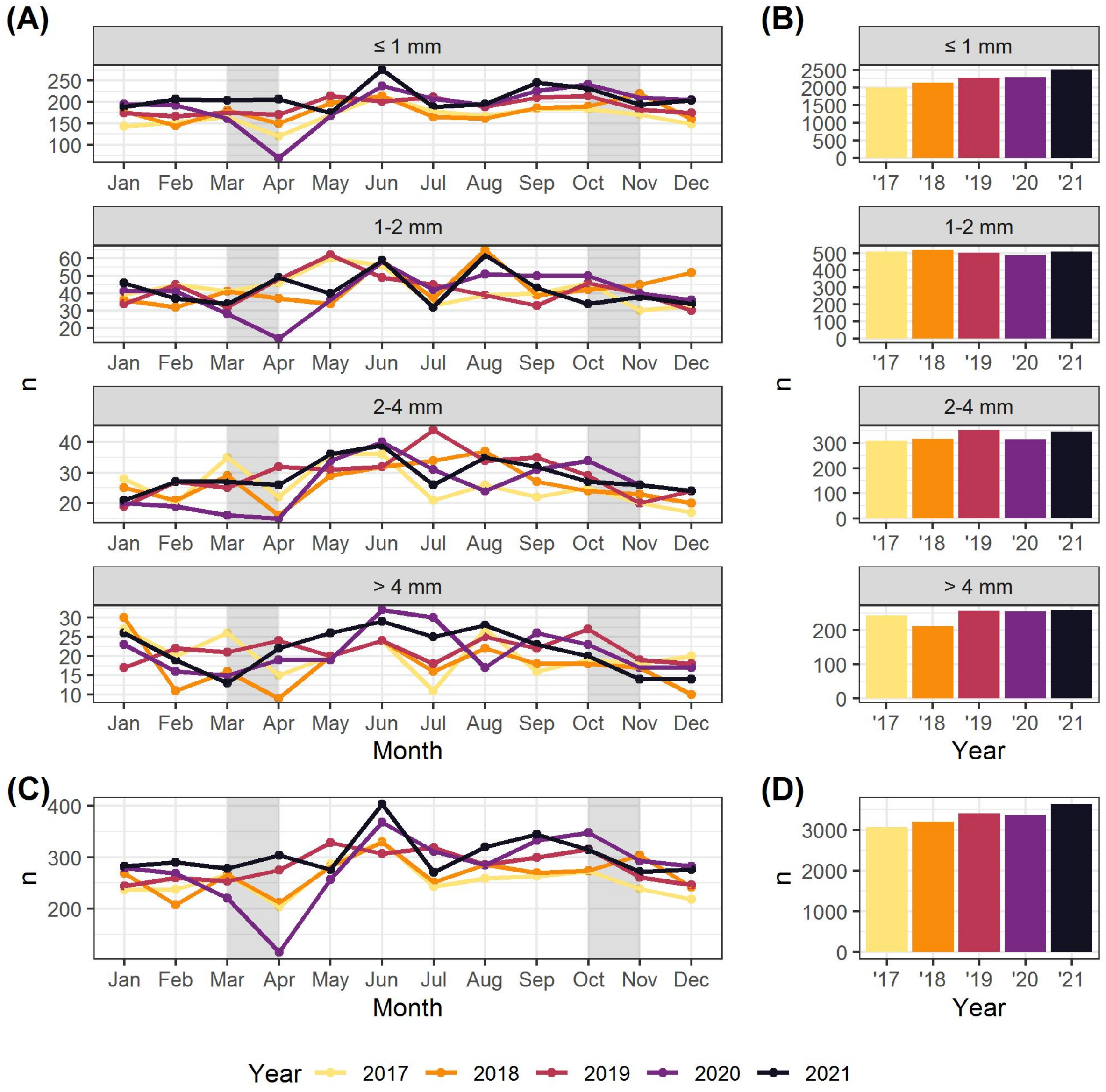

Figure S2. Evolution in monthly (A) and annual (B) number of melanoma diagnoses, broken down according to predefined Breslow groups ([0,1],]1,2],]2,4],]4,infinite]). The grey areas correspond to the periods in 2020 in which an exponential growth in the number of COVID-19 infections was observed.

Table S1. Absolute and relative difference in number of cutaneous melanoma diagnoses compared to the previous year, by Breslow thickness.

	**Breslow subgroup**	**2017**	**2018**	**2019**	**2020**	**2021**
Absolute number of diagnoses	[0,1[	1999	2147	2284	2307	2517
	[1,2[	508	519	503	487	508
	[2,4[	308	317	352	314	346
	[4, ∞[	243	211	257	254	259
	**Total**	**3058**	**3194**	**3396**	**3362**	**3630**
Absolute difference in number of diagnoses compared to previous year	[0,1[	n.a	148	137	23	210
	[1,2[	n.a.	11	-16	-16	21
	[2,4[	n.a.	9	35	-38	32
	[4, ∞[	n.a.	-32	46	-3	5
	**Total**	**n.a.**	**136**	**202**	**-34**	**268**
Relative difference in number of diagnoses compared to previous year	[0,1[	n.a.	7.40	6.38	1.01	9.10
	[1,2[	n.a.	2.17	-3.23	-3.18	4.31
	[2,4[	n.a.	2.92	11.04	-10.80	10.19
	[4, ∞[	n.a.	-13.17	21.80	-1.17	1.97
	**Total**	**n.a.**	**4.45**	**6.32**	**-1.00**	**7.97**
